# Learning From Love Island? Diversification of the Hegemonic Man

**DOI:** 10.3389/fsoc.2019.00072

**Published:** 2019-11-12

**Authors:** Kitty Nichols

**Affiliations:** Department of Sociological Studies, University of Sheffield, Sheffield, United Kingdom

**Keywords:** Love Island, masculinity, emotion, care, gender performance, reality TV

## Abstract

It is undeniable that Love Island promotes specific ideas of masculinity and masculine behaviors. There is an “expected” masculinity performed in the villa, exemplified in cases, such as “The Do Bits Society” which advocates heteronormative forms of masculinity and gender relations (Whitehead and Barrett, 2008). Within such examples men had to successfully perform what Schrock and Schwalbe (2009) refer to as “manhood acts” in order to prove their masculine identity. This form of masculinity, which dominated the space, can be explained sociologically via intersecting hegemonic and performance theorizing (Goffman, 1974; Connell, 2005; Butler, 2008; Wellard, 2009). However, utilizing new combinations of theoretical approaches, this paper will explore more diverse performances of masculinity present in the villa. This includes the ways that men were making choices in the construction of their masculine identities beyond the “expected” masculinity which dominated, as well as how women also performed this form of masculinity. Through analysis of two seasons of Love Island (2018 and 2017), this paper will highlight how lines between different ways of living and experiencing masculinity can be blurred and fluid. In doing so, the paper encourages a critique of how we theorize masculinity and gender more widely, allowing for emergent theorizing which blends existing theories in new ways.

## Introduction

The genre of reality TV has become a popular part of culture and includes shows which document diverse aspects of the everyday world and interactions (Sears and Godderis, 2011; Negra et al., 2013). Related to this, is the importance of the “celebrity” to popular culture, specifically, the ways in which celebrity discourses weave into public imagination (Feasey, 2008; Allen and Mendick, 2013). According to Feasey (2008), the “explosion” of media genres including that of celebrity and reality TV demonstrates that this is an important area of research, with individuals forming bonds with other consumers and creating what Bird (1992) refers to as “imagined communities” via the consumption of these forms of media. Further to this, the appeal of what Andrejevic (2003) refers to as “the real” explains the sustained interest in this media genre. Reality TV's ubiquity and prominence has made it a site which is significant to study, acting as a lens through which to understand and extend knowledge of long established fields, such as gender.

Previous work has outlined the significance of media to understanding the maintenance and dominance of certain groups and structures within society (Biressi and Nunn, 2005; Hill, 2014). Gramsci (2005) refers to this authority as hegemony, originally developed in relation to class, however subsequently adopted to explore gender relations. Hegemonic masculinity, as outlined by Connell (2005) utilizes Gramscian concepts to understand how men have sustained a leading position in society. Notably, within much of the literature discussing Gramscian perspectives of hegemony, emphasis is placed upon persuasion of dominant cultural ideas through the media, resulting in ideologies being normalized (Donaldson, 1993; Demetrious, 2001; Thompson, 2002; Connell, 2005). Whilst hegemonic masculinity proves slippery to understand, application to new areas, such as reality TV, produce additional dimensions to existing debates (Donaldson, 1993; Howson, 2006; Pringle, 2008).

Work on meanings and pleasures afforded by reality TV and related media has increasingly come to the attention of academic debate, however, specificity in terms of understanding the case study of Love Island is yet to be explored (Turner, 2004; Holmes, 2005; Feasey, 2008). Love Island is a reality TV dating show set in Mallorca Spain, in which contestants live in a luxury villa where they are constantly under surveillance. The contestants are isolated from the outside world, living and socializing together for up to 8 weeks. To survive in the villa, all contestants must be in a couple, with recoupling and eliminations occurring throughout the series. The overall winning couple, voted by the public, receive £50,000. The show invites audiences to watch and moralize about the contestants, who are largely young, heterosexual, working class individuals. In doing so, exacerbating societal inequalities and arguably exploiting participants for the public gaze (Esoffery, 2006; Squires, 2008). Additionally, Love Island promotes a specific type of aesthetic, depicting masculinity as being tied to the body, with toned, athletic and strong bodies framed as desirable. Emphasis placed upon bodies within the series leads to a particular body type being expected or required, with producers selecting contestants based on modern gender ideologies that exacerbate gender differences (Connell, 1995; Turner, 2001). Furthermore, the format of the show, which is highly edited, creates a selective portrayal of contestants and skewed power dynamics, favoring audiences rather than contestants.

Whilst there has been a growing body of work exploring gender, class and ethnicity more broadly in relation to reality TV, focus on masculinities within this field remains underdeveloped (Allen, 2011). In terms of gender, previous work has placed emphasis upon exploring femininity and the ways in which reality TV constructs and problematizes femininity (McRobbie, 2004; Allen, 2011). Elsewhere, existing work on men and reality TV has tended to focus upon examining the intersection between media and sexuality, exploring how reality TV provides a space for more diverse forms of masculinity to become embedded within the public imagination (Alderson, 2014).

This paper builds on existing work surrounding the construction of identity, utilizing the 2018 and 2017 seasons of Love Island as a case study to explore the way that individuals “do” gender within the villa (West and Zimmerman, 1987; Jenkins, 2004). Data for the paper comprised viewing and analysis of 90 episodes of Love Island over the 2018–2017 seasons. Visual methods, allowing for exploration of how visual materials indicate underlying social factors, specifically gender and sexuality were implemented (Alexander, 2011). Analysis comprised of content analysis, enabling the communication and representation of gender and sexuality to be explored (Alexander, 2011; Babbie, 2013). Content analysis followed two phases: firstly, descriptive analysis of the episodes, summarizing data and identifying main trends (Sarantakos, 2013). This initial phase also enabled identification of episodes for viewing in phase two. The second phase involved exploratory analysis, whereby episodes were analyzed more closely in relation to the theme of masculinity. Feminist and interactionist approaches to research were adopted, allowing gender to be viewed as a social construction, thus enabling a critique of masculinity (Hesse-Biber, 2012; Pini and Pease, 2013). As noted above, the producers' agenda and highly edited format of the show arguably limits the ways that contestants are able to “do” gender, however this remains a useful case study to explore intersections and tensions within contemporary theorizing on masculinity.

Utilizing the lens of performance outlined by thinkers, such as Goffman (1959) and Butler (2008), alongside gender theorizations including hegemonic and inclusive masculinities, this paper will act as a catalyst for considering new theoretical directions in the study of men's lives (Connell and Messerschmidt, 2005; Wellard, 2009; Magrath, 2017; Anderson and McCormack, 2018). Whilst tensions between various writing on gender utilized here must be acknowledged, this paper does not seek to provide a new theory for studying men's lives, rather it aims to act as a catalyst for more nuanced debates within masculinity scholarship. For example, through analysis of gendered interactions and behaviors within this reality TV show the case will be made that diverse gendered performances occur. More specifically, this paper will challenge dominant typologies of gender configurations, such as hegemonic masculinity, utilizing analysis of men displaying emotion and care, as well as the performances of masculinities by women, as a way to illuminate the limitations with current theorizing. Via examining the 2018/2017 seasons, the paper will demonstrate that the boundaries of gendered identities are changing, with implications for how gender is understood beyond this media genre.

## Masculinity and Performances of Manhood

To understand and explore diversity in masculinity, it is first important to extend the discussion of theorizing on hegemonic masculinity outlined above, as this dominant theorizing underpins much of the scholarship on masculinity (see Connell, 1995, also Connell and Messerschmidt, 2005). Theorizing on hegemonic masculinity continues to be a prominent paradigm through which gender configurations including masculinity are understood. Additionally, the link between hegemonic masculinity and heterosexual masculine identity is prominent. This is particularly the case when discussing collective male behaviors and so called lad cultures, in which heterosexuality is often performed, expected and celebrated (Jackson, 2002; Pringle, 2008; Dempster, 2009; Jackson et al., 2014). Whilst the definition of hegemonic masculinity has become contentious, due in part to the wide usage of the term, hegemonic masculinity continues to be widely cited in research on masculinities (Connell and Messerschmidt, 2005; Howson, 2006; Magrath, 2017; Roberts, 2018). Consensus remains that the term is understood in relation to Connell's initial theorizing, referring to “the pattern of practice that allowed men's dominance over women to continue” (Connell and Messerschmidt, 2005, p. 832). The continued relevance of hegemonic masculinity theorizing has been discussed, with Hearn (2004) acknowledging that it remains a significant tool to adopt in the study of gender, asserting that the idea helps us to understand the ways in which men dominate both women, and other men (see also Moller, 2007; Roberts, 2018).

Hegemonic masculinity is not a fixed formula and is not being advocated as such in this paper, rather it is a configuration of practices which are tied to organization and institutions, including media and reality TV (Donaldson, 1993; Connell, 2005; Connell and Messerschmidt, 2005; Howson, 2006). Theorizing on hegemonic masculinity can thus be a tool to see and understand the ways that masculinity has become dominant, more specifically, the ways in which particular characteristics have become synonymous with gender ideologies (Connell, 1995; Thompson, 2002). Reality TV is one such sphere of society where hegemonic masculinity continues to be depicted and reproduced. A clear example of this can be seen in the case of Geordie shore (UK version) and Jersey Shore (US version) in which heterosexual masculinity encompassing characteristics, such as risky behaviors, no emotion, toughness and idealized body types are promoted and normalized. Here audiences are shown ideal types of masculinity, with characteristics, such as heterosexuality, toughness, strength and virility celebrated. This supports existing work which states that all men experience masculinity as part of their everyday lives and are often unaware of it, with implications for other individuals and groups within society (Messner and Sabo, 1990; Hearn, 2004; Robinson, 2008; Wellard, 2009).

More recently there have been challenges to the concept and structures of hegemonic masculinity, including examination of themes, such as choice, critical consciousness and resistance (Jewkes et al., 2015; Messerschmidt et al., 2018). This paper will continue to explore some of these themes, utilizing the example of Love Island as a catalyst for new debates on masculinity scholarship.

A further conceptual tool which provides a foundation to begin unraveling masculinity in Love Island, is that of performance. This lens presents an explanatory framework for understanding identity (Gutterman, 2008). Jeff Hearn acknowledges the relevance of performance in the construction of masculine identities in the following quotation from his work on men and masculinities:

“*Masculinities are not fixed formulae, but rather combinations of actions, part powerful, part arbitrary, performed in reaction and relation to complex material relations and emotional demands, and recognised by others as signifying that this is a man” (Hearn*, *1994**, p. 54)*.

Building on this, it is argued that becoming a man is a dramaturgical task and that men need to perform manhood acts to prove masculine identity (Schrock and Schwalbe, 2009). Performance also provides a way of thinking about the social world, evidenced in work which utilizes performative language and frameworks in order to develop arguments around the topic of identity (Curry, 1991; Cameron, 1992; Gutterman, 2008; Walsh, 2010). Erving Goffman's conceptualization of the dramaturgical theme in his work *The Presentation of Self in Everyday Life* (1959), in which he notes that people consciously present themselves to others through performances, is one of the earliest works on performance and heavily influenced subsequent work on interactions and identity. Thinking specifically about gendered identities, performance is a recurring theme within this literature, though is often underplayed, utilized as a conceptual tool in order to engage with the various ways that gendered identities are formed and negotiated (Cameron, 1992; Kimmel, 2006; Butler, 2008; Walsh, 2010). The idea of performing masculinity is common within literature, as well as popular media representations, such as Love Island. Within reality TV including Love Island, notions of masculine performances are embedded within discourses, practices and symbols; conveying traits, such as aggression, strength, virility and mental toughness (Mac an Ghail and Haywood, 2007). Within this paper performance will be connected to hegemonic masculinity in order to provide a framework for critical thinking regarding masculinity in Love Island.

## The “Do Bits Society” as Islander Hegemony

Within the 2018 series of Love Island viewers watched as the men established a group which they named the “Do Bits Society” (DBS). This was an exclusive group, comprised of men only, whereby full membership was granted once an intimate sexual act with a female contestant had been undertaken. Further to this, the group established “levels” in order to police entry. This included the creation of a ranking system comprising different intimate interactions, ranking these according to perceived significance, with penetrative sex valued most highly and kissing the least. Entry to the group was celebrated and those who were seen to be taking excessive amounts of time to enter the group, such as Dr. Alex, were routinely ridiculed and placed under pressure to gain entry. The group reflected the underlying emphasis and value placed upon heterosexual success which permeates the show (across both seasons). The form of masculinity associated with the DBS became the dominant masculinity in the villa, in doing so becoming the hegemonic masculinity in the space (Connell, 2005, Connell and Messerschmidt, 2005). This example provides an interesting starting point from which to examine the various performances of masculinity which were present in the villa. More specifically, this case enables an exploration of the choices and agency men displayed when negotiating their masculinities within the everyday of villa life.

The “Do Bits Society” can be understood via the lens of performance and hegemonic masculinity theorizing. This society became an exclusive form of masculinity in the villa, developing into the localized hegemonic masculinity within the small community, requiring sustained performances of specific behaviors and interactions (Wellard, 2002, 2009; Connell and Messerschmidt, 2005; Messerschmidt, 2012). The DBS dominated alternative masculine performances and, as will be explored later in the paper, was eventually mirrored by women. The way that this masculinity permeated all interactions in the villa reflects analysis of hegemonic masculinity by thinkers, such as Hearn (2004) and Connell (2005), in that it relied upon collective actions, collusion and complicity in order to continue.

This resonates with the work of Schrock and Schwalbe (2009), who assert that in order to be deemed as a “man,” men must prove themselves within their everyday lives, undertaking actions which align with expected or hegemonic form of masculinity to do so (Wellard, 2002, 2009; Connell, 2005; Whitehead and Barrett, 2008). The contestant Wes was the first to gain entry to the society, establishing himself as the founder, after undertaking sexual acts with Laura. He noted “*Until one does bits, one can not have the password to the Do Bits Society Club*.” His success with Laura was applauded by men in the villa, with audiences of the show viewing the men gather away from the women immediately afterwards to discuss the minutiae of the activity. Significantly here, Wes was recognized by his fellow male counterparts as being a “legend” or “hero” and given status based on his performances of manhood acts (Schrock and Schwalbe, 2009).

Wes's successful performance of masculinity was enabled by the collusion of other men and specifically, the continued recognition and celebration of such masculine performances beyond the first DBS meetings. Whilst not all men were part of the group, membership remained coveted. For example, Jack Fincham was not able to join the group due to the strong views surrounding sexual intimacy in public shared by his partner Dani Dyer, yet he remained associated with the group and celebrated the achievements of other members. The work of Gregson and Rose (2000), viewed alongside Goffman (1981), can be adapted to explain this example, acknowledging that performances are important in the processes of understanding and establishing the everyday rules and routines which compromise the social world. Further to this, performances in everyday lives become so much part of the routine that they are no longer questioned. This work illuminates the importance of the DBS in constructing and reinforcing the dominant masculinity in the villa, as in order for the DBS to operate, it required other men to engage and uphold its values without question. Despite the fact that Jack was not a full member, nor likely to be, he continued to follow the norms established by the DBS. In doing so, he colluded to show support and ensure inclusion within the group.

The importance of the DBS to the construction of the dominant form of masculinity in the villa was exemplified in instances where men failed to become members. The performances of masculinity associated with the DBS echoed what Wellard (2009) refers to as “expected” masculinities, whereby men were anticipated to display particular characteristics aligning with the dominant model of gender relations in the space. According to Wellard (2009), men have to work in order to “fit in” with dominant forms of masculinity. In doing so, there is the implication that this is a conscious decision and that those men therefore have the potential to deviate, or make different choices, with regards to how they situate themselves within the gender order (ideas which will be explored in more detail later in the paper). A clear example of this was in the case of a contestant called Dr. Alex, who outside of the villa was a medical doctor, working in an emergency department. In the 2018 series he was famed for being unlucky with the ladies and as a result of this, was often close to being evicted from the island. To remain in the villa, Dr. Alex formed close friendships with women, utilizing friendship as a form of capital and to ensure popularity. Significantly, Dr. Alex possessed other qualities, or forms of capital which in the outside world would situate him at the top of gender configurations of hierarchies, for example he possessed status and class privilege which contrasted with the working class backgrounds of other contestants (Hearn, 2004; Connell and Messerschmidt, 2005). However, within the villa, his inability to translate this capital to success with the women meant that he was perceived to be a failure (Bourdieu, 1986, 1993; Connell and Messerschmidt, 2005; Howson, 2006).

An interesting point in the villa which demonstrated the proliferation of the DBS occurred toward the end of the series when Dr. Alex “coupled up” with Alexandra. Here we saw the pressure of performing expected masculinity and undertaking actions associated with the DBS clearly displayed (Wellard, 2009). After a few dates and nights sharing a bed, the men were keen to see whether Dr. Alex would be permitted entry to the DBS. Significantly, Dr. Alex, who up to this point had been framed by women in the villa and viewers as a “nice guy” due in part to his chivalry and respect for women, was seen describing an intimate interaction with Alexandra, which involved him showing her his genitals. Whilst this was judged by his peers as not counting as sufficient interaction to gain full entry to the DBS, the men celebrated this progress. The significance of this case, was that viewers framed this behavior as inappropriate, positioning this in stark opposition to his “true” identity (Jenkins, 2004). This was due to the fact that Alexandra had been so shocked, noting to the women in the villa that these sexual advances were uninvited and unwanted. Viewers believed that Dr. Alex had succumbed to the dominant male behaviors performing masculinities which were expected of him. When probed about his interaction with Alexandra in the villa diary room by producers, Dr. Alex admitted feeling pressure to be successful with her as he “didn't want to let the guys down.” This example clearly highlights the influence of hegemonic masculinity in the space and that in some cases, men experience pressures to conform.

## Diverse Masculinities: Demonstrations of Choice and Resistance in Villa Life

Connell's (2005) concept of the gender order and hegemonic masculinity center upon the notion of power, with the assumption that men are complicit within the maintenance and continuation of hegemonic gender structures and dynamics within society. This idea has been illuminated in the previous section via the discussion of the DBS, whereby the dominant masculinity dictated behaviors and interactions within the villa. However, whilst hegemonic masculinity remains an important tool to adopt in the study of men and gender more broadly, critical examination of the theory uncovers faults (Hearn, 2004; Waling, 2019). Building on thinking from writers, such as Connell (2005), which indicates multiplicity and contradictions in masculine identity formation, theorizing which illuminates more diverse forms of masculinity has emerged (Bryson, 1990; Anderson, 2012; Magrath, 2017). Such recent theorizing captures themes including resistance, choice and transgression (Anderson, 2012; Jewkes et al., 2015; Magrath, 2017).

Seidler (2006) argues that hegemonic masculinity theorizing limits the capacity for acknowledgment of men's subjectivities, experiences, practices and possibilities for change (see also Waling, 2019). Seidler (2006) suggests that thinking in terms of the gender order is too rigid, proposing that this misses diverse aspects of masculinity including the expression of emotion. Hanlon (2012) assesses convergences within theorizing from Connell and Seidler, asserting that thinking about either power or vulnerability in a vacuum is problematic, arguing instead that dominant forms of masculinity and values of emotion and care are not antithetical to one another. Rather, understanding these as interwoven allows for a more in depth analysis of masculinity. The following section will utilize this framework as outlined by Hanlon (2012) to consider diverse masculinities within the context of Love Island. Specifically, it will highlight the ways in which men actively make choices to show emotion or values of care, and in doing so, demonstrate resistance to the DBS or hegemonic masculinity which dominated the space.

From the examples presented so far, it is clear that Love Island, along with other reality TV shows, such as Geordie Shore and The Bachelor act to reassert notions of hegemonic masculinity which permeate British culture. These examples celebrate forms of masculinity which position men who are successful with women, physically strong and mentally tough as superior to others and “true men” (Connell, 2005). This reflects the belief in popular culture that there is a fixed formulae of masculinity and that men “do” masculinity a particular way, overlooking the nuances of male experiences (West and Zimmerman, 1987; Connell, 2005; Robinson et al., 2011; Elliot, 2015). Whilst the landscape of masculinity has undoubtedly changed, with writers including Magrath (2017) and Anderson (2012) indicating that diverse masculinities are possible, this requires further analysis. Indeed, the interpretations and prominence of hegemonic masculinity outlined in the previous section remain accurate; however, arguably, there are more diverse forms of masculinities present within Love Island which warrant exploration. This includes masculinities that emphasize displays of emotion and care.

The diversification of masculinities has become an increasing focus of research. For example, Barrett (2008) in his work on men in the US Navy, asserts that the image of masculine hegemony which characterizes men as physically tough, aggressive, unemotional and heterosexual is not permanent. Anderson (2012) a key critic of the hegemonic model of masculinity adds to this, noting that the esteemed versions of masculinity are changing. Anderson (2012) goes on to argue that “inclusive masculinity” more accurately depicts men's experiences, with men now able to behave in ways once associated with homosexuality, including display of emotion, without experiencing a threat to their public identity. This alternative theoretical framework which explores diverse gender relations and performances has important implications for how masculinity can be understood within Love Island.

A significant facet of inclusive masculinity theorizing, is the suggested shift toward a less rigidly vertical notion of gender hierarchy, with macho behavior denigrated and “softer” masculinities valorized (Anderson and McGuire, 2010; de Boise, 2014). This acknowledges the presence of multiple masculinities, with the potential for versions of masculinity to be situated alongside one another as opposed to “on top” (Connell and Messerschmidt, 2005; de Boise, 2014). Exploration of multiple masculinities has most commonly been examined within hypermasculine sites including sport, where diverse masculinities have been less common (Tsay-Vogel and Krakowiak, 2017). An example of such work is by Crocket (2012) and his research on Ultimate Frisbee. Crocket (2012) provides an analysis of the way that participants in his study performed a range of diverse masculinities within one Ultimate Frisbee season. In doing so, Crocket (2012) suggests that one stable version of masculinity is not possible, rather men transition through and between versions of masculinity. This work builds on research by Robinson et al. (2011) on masculinities in transition, where they argue that male identity is processual, always under negotiation and the outcome of ongoing performance.

Extending such ideas to the men in Love Island, it can be argued that over the course of the 2018 series in particular the gender configuration shifted, with the versions of masculinity which were prominent at the start changing and men performing more diverse forms of masculinity (Markula and Pringle, 2006; Crocket, 2012; de Boise, 2014). The following sections explore these ideas further, starting by examining the idea of the emotional man, followed by discussion of caring men. Notably, it is important to acknowledge that contestants are continually in the process of managing their identity in order to win the show, therefore, the presentation of self and gendered performances might not be authentic. However, these examples remain important stimuli to probe existing masculinity theorizing.

### The Emotional Man

Reality TV often emphasizes the expression of emotion, documenting the “emotional journeys” of characters or contestants. This is exemplified in shows including The Bachelor, The Island and Big Brother, in which episodes routinely include an individual becoming openly emotional. This outpouring of emotion has been termed elsewhere as the “money shot,” recognized as being utilized by producers to draw in audiences (Grindstaff, 2002; Dubrofsky, 2009). Most frequently, we see women positioned at the center of scenes of emotional disclosure, or undertaking emotional labor, with less attention given to men (McQueen, 2017). Within Love Island, there is evidence to suggest that women are still being depicted as those most likely to convey emotion. For example, in the numerous occasions where the female contestants are presented crying in the diary room, or the instances where women comfort each other after dramatic recoupling events. However, what is interesting, is that the men in Love Island are experiencing emotion in a myriad of ways, in doing so beginning to rupture existing understandings of men within this TV genre and showing resistance to the dominant gender structures.

Viewing men as emotional has become more widely accepted, with emotional disclosure and expression seen to be present in even the most hyper masculine spheres including sport (Muir and Seitz, 2004; Robinson et al., 2011; Campenhout and Hoven, 2014). The notion that men should be more emotional is not new, credited to the rise of the feminist agenda (Seidler, 1997). However, there have been adverse reactions to this on the basis that this leads to further “emotional insecurities” experienced by men (de Boise, 2015:3). Regardless of the critique, the idea that “it's good to talk” has remained a prominent discourse resonating throughout society, reflected in the rise in mental health campaigns in the UK including Movember^1^ and CALM^2^ (Illouz, 2008; McQueen, 2017).

As noted above, notions of hegemonic masculinity are traditionally built on a system in which men's expression of emotion is downplayed or hidden, with previous studies demonstrating that men's emotional inexpressiveness was influenced by patriarchal privilege (de Boise and Hearn, 2017; McQueen, 2017). Recent work has acknowledged that men are aware of their emotions, with expression of emotion being more widely accepted (Lilleaas, 2007; Anderson and McGuire, 2010; de Boise and Hearn, 2017; Nichols, 2018). Studies of emotional disclosure have begun to explore the tensions between the cultural discourses surrounding how to be “manly” and how to “do” emotion. In her work on emotional expression of men in their intimate relationships, McQueen (2017) notes that men value emotional disclosure, experiencing vulnerability associated with disclosure due to popularized narratives, such as “boy's don't cry.” Further to this, McQueen (2017) argues that men desire to be emotionally fluent in interactions and relationships, however feel this is challenging due to fear of exposure through sharing their vulnerability. This work is important to consider in relation to the men in Love Island, as intimate relationships normally occurring in private spaces, unfold in the public eye. Arguably, this compounds the pressures to conform to dominant or expected discourses of masculinity, such as that advocated by the DBS, in order to appear “manly,” as exemplified in the previous discussion of Dr. Alex (Wellard, 2009).

One clear example from the 2018 series which demonstrated that men are able to resist the dominant gender structures and express emotion openly, though continue to feel in some ways restrained by expected masculinity, was in the case of Jack Fowler, when he discussed his experiences of breaking up with a female partner outside of the villa. Below is a transcript of his conversation with other contestants, followed by his private and continued reflections from within the villa diary room.

*Jack: “Every time you fall in love or something it is a different type of love and when it ended I was heartbroken yeah. I was like brah*^3^ *what's this pain man, I was like what kind of pain is this?”*

Josh: Mate its bad

Jack: It is nothing physical, its emotional, it can make you be ill. I didn't eat, I lost weight, I was crying all the time

Paul: It is sort of funny how you notice that every song that comes on the radio is something to do with like a break up

*Jack: Uh huh, mate it enhances you so much*.

Jack diary room reflections:

*I think especially being a guy, sometimes it is hard to, um voice how you feel. I have been through heartbreak, I have gone through that and yeah it is not nice. So yeah, talking to the others about it was quite refreshing actually, because guys, we don't really do that*.

(Season 4, Episode 49: 22nd July 2018)

The reaction to this interaction and the disclosure of his emotions was praised by viewers, with many suggesting that Jack had “quashed” the idea of toxic masculinity^4^ (Gordon, 2018). Significantly, the public reaction to this, understood alongside Jack's narrative, enables the possibility for diverse forms of masculinity to be further explored. Within this interaction, Jack is attributing his reluctance to disclose how he felt to his fellow islanders to the pressures and expectations of masculinity. As a semi professional footballer, Jack was arguably accustomed to performing what Wellard (2002, 2009) refers to as “expected” forms of masculinity. The acknowledgment here from Jack that disclosing his emotions so openly was a “*refreshing*” experience demonstrates that men remain vigilant in the articulation and expression of emotion. This also exemplifies the role of space in emotional disclosure, as Jack was only completely open in the diary room, away from the other men. Despite remaining constraints, this example shows that the men found ways to convey emotion and to negotiate the structures which limited their behaviors.

**Figure 1:**
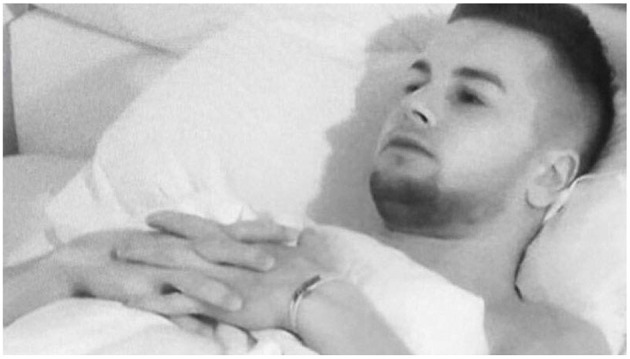
Image of Chris Hughes during season 3.

A further example of this resistance to hegemonic masculinity in the villa, can be seen in the 2017 series where islander Chris Hughes openly discussed his struggles with mental health issues and frequently expressed his emotions whilst in the villa. During his time on the show Chris had a volatile relationship with Olivia Attwood, resulting in him crying after tense interactions and arguments. Scenes of Chris crying in the diary room, or in bed at night, became staples of every episode. This spawned memes and GIFS of Chris crying which proliferated social media (see Figure 1, below). However, despite the public caricature of this image, the overwhelming response was that Chris was praised publicly for “bearing all” (Ojomu, 2017). What was interesting about this example is that as viewers, we saw him continue to grapple with balancing different facets of his masculinity. This resonates with the idea that men struggle to “do” emotion and to navigate this alongside expectations of characteristics associated with the hegemonic male (Kiesling, 2005; de Boise, 2015). However, whilst Chris did not always manage to find this balance perfectly, the example and public reaction demonstrates that there is potential for more dynamic and diverse forms of masculinity to operate within this space and that these would be accepted. This extends the work of de Boise (2014) indicating that masculinity is multiple and that versions of masculinity are situated alongside one another, as opposed to one dominating the other, with men able to transition between these with fluidity and an awareness of this shift.

These two examples suggest that utilizing hegemonic masculinity alongside conceptions of performance as a lens through which to understand the men in Love Island exposes tensions and limitations of Connell's (2005) gender order theorizing. Whilst hegemonic masculinity in the form of the DBS persists, understanding the men through this lens alone is problematic, missing the nuance of masculine performances and experiences. Through exploring the ways in which men are conveying emotion in the villa, it is evident that masculinities are multiple and that men are able to make choices and transition between different facets of masculinity, in doing so extending Connell's work and advocating alternative ways of theorizing men's experiences which accounts for diverse performances.

### The Caring Man

In order to add depth to the critique of hegemonic masculinity being proposed within the paper so far, and to continue exploration of alternatives, it is beneficial to address emotion more specifically. Existing sociological and psychological work on singular feelings characterize “feminized” emotions as including care, sympathy and emotional display (Smiler, 2004; Chodorow, 2014; Patulny et al., 2017). Whilst emotional display has been discussed in the previous section, here the notion of care, specifically the value men in Love Island placed upon this, will be the focus of the analysis, once again enabling the potential for more diverse and multiple facets of masculinity to be explored.

Hegemonic masculinity provides a cultural reference point for men, which can act to limit behaviors and interactions within particular spaces (Hearn, 2004; Coles, 2009; Elliot, 2015). Arguably, this is the case in relation to men displaying values of care, where there are barriers to men's participation, stemming from the sense of power and entitlement hegemonic masculinity holds (Kimmel, 2010; Elliot, 2015). Elliot (2015) in her work exploring caring masculinities, has begun to rethink the ways in which men understand masculinity, asserting that through examination of the ways in which men display values of care, we are able to see changing conceptions of masculinity.

Elliot (2015) aims to reconceptualize masculinity theorizing, arguing that men are rejecting the values associated with domination, instead showing care and emotion in order to work toward gender equity. Within this reconceptualization of masculinity, Elliot (2015) recasts traditional masculine values including “protection” and “providing” into relational, care-oriented ones. For example, competence within this case does not refer to mastery over another, or of a skill; rather this refers to the ability to care (Morrell and Jewkes, 2011; Hanlon, 2012; Elliot, 2015). Whilst much of Elliot's work is based on work with families, the context of the villa and the imitation of a family, enforced via proximity, formation of close bonds and isolation from those in the outside world, creates a family of choice (Weeks et al., 2004). Thus, Elliot's (2015) work can be appropriately applied to the analysis of men in Love Island in order to create new insights into the diverse performances of masculinity.

Hochschild (2012) describes “feeling rules” where social norms dictate how we believe we should feel and act. This idea is often utilized to explain the structural differences in men and women's behaviors, particularly in terms of roles individuals are expected to play within social interactions (Patulny et al., 2017). Further to this, historically women and men who display gender incongruent emotions have been challenged or received social disapproval (Patulny et al., 2017). In relation to men, this group would be categorized as “marginalized” or “subordinated” masculinities (Connell, 1995; Ralph and Roberts, 2018). Within Love Island, there are clear examples of men undertaking caring roles, in doing so, blending gender roles and re-categorizing marginalized and subordinated masculinities. The most pertinent example of this is the introduction of baby dolls, where each couple are given baby dolls to look after in order to simulate being parents in the outside world. These dolls are interactive, manipulated by producers to cry, scream and laugh to emulate real babies. The task involves the couples successfully caring for the babies, which includes feeding, bathing, comforting and dressing the babies appropriately, with those deemed to be the “best parents” rewarded at the end of the task. Notably, the baby has to be cared for by both parents in order for the couples to successfully pass the task.

This task has been implemented over the last few series, becoming a popular section of the show, framed as “making or breaking” couples. Within the 2018 series we once again saw the arrival of the baby dolls, with intriguing effects on the men and performances of masculinity. During this period the female islanders were sent away from the villa, leaving the men in charge. This signaled Wes branding this change in the men's circumstances as a rebranding of the DBS, he noted: “*The DBS Society has now become the Daddy Daycare Society, the DDS*.” The acknowledgment by Wes of this shift once again shows that men are aware of their behaviors and the parameters under which hegemonic performances are acceptable.

The task revealed a “softer” side to many of the men, with emphasis placed upon care and nurturing, evidenced in the commitment to looking after the dolls. In doing so, aligning with arguments which suggest that more inclusive forms of masculinity exist in hyper masculine spaces (Anderson, 2012; Magrath, 2017, Nichols, 2018). This was seen most clearly in instances where the men were left alone with the dolls, during which time viewers saw men showing affection and empathy, with these characteristics dominating their performances of masculinity, facets which had been downplayed at other points of the series. In doing so, the masculine characteristics which they had been demonstrating most frequently were challenged, showing ruptures in hegemonic masculinity, also highlighting the capacity for men to transition between these identities quickly (Connell, 2005; Robinson and Hockey, 2011).

The ability for men to make these quick transitions within their performances of masculine identities was evidenced most clearly when the women temporarily exited the villa, leaving the men to care for the dolls. At first the men cared for the dolls diligently, however, viewers watched as the men proceeded to conduct a pram race, resulting in the dolls being catapulted into the air, actions which had the dolls been real, would have been catastrophic. Immediately after this event the men were seen to show concern for the dolls, transitioning immediately back into “caring mode.” This lapse from caring mode back into the hegemonic display of mischievousness was interesting, extending the work on transition and multiplicity outlined so far. Here we see that the men were able to convey diverse performances of gender almost simultaneously, doing so knowingly and moving between these with ease. Patulny et al. (2017) note that it is important to acknowledge the flow and continuity between different feelings which men might experience simultaneously (see also Coles, 2009; and Nichols, 2018). However, knowledge surrounding the agency and immediacy with which this happens remains underdeveloped.

Previous work has tended to suggest that men perform either hegemonic or alternative masculinities, overlooking men's agency within these transitions, or the possibilities that men might be able to do multiple simultaneously (Light and Kirk, 2000; Kimmel, 2010). Whilst there are some exceptions, with research demonstrating that men's identities transition across the life course, attention to the processes and immediacy of transitions has been overlooked (Coles, 2009; Robinson and Hockey, 2011; Patulny et al., 2017). This Daddy Day Care example aligns with the work of Bridges and Pascoe (2014), who suggest that men can demonstrate “hybrid masculinities,” whereby bits and pieces of masculinities and, at times femininities are conveyed. Whilst this work illuminates the blending of gendered performances and highlights gender as a project, the examples from Love Island act to extend such ideas, starting to unravel the ways that men do this within their everyday lives, highlighting the process of transition more closely (see also Nichols, 2016).

The examples of men showing emotion and care within this section have demonstrated that men are able to display facets of their masculinity simultaneously, in doing so resisting rigid hegemonic structures and demonstrating the capacity for choice. This echoes feminist perspectives on agency, whereby this is understood according to Waling (2019: p. 99) “as the capacity for one to act in a particular environment.” Viewed alongside the work of Ralph and Roberts (2018), who note that hegemonic structures are often weakened rather than completely transformed; it can be argued that whilst hegemony is an undercurrent within interactions and identity construction, more diverse forms of masculinity are present and men are able to display these with relative ease.

## Blurred Performances

Connell (1995) argues that those who are marginalized, or subordinated by hegemonic masculinity are “complicit” in the continuation of this dominant structure. However, the question remains of what happens to those who enact the hegemonic masculinity archetype, yet do not benefit from it? The confines of the Love Island villa enables the possibility for what Connell (2005: p. 72) refers to as “gender projects” to be undertaken, whereby gender is constructed and negotiated within the parameters of the expected gender structures. So far, this paper has explored the ways in which men have engaged with hegemonic masculinity, resisting and challenging this, in doing so illuminating that typologies of masculinity are problematic, as they do not account for diverse masculine performances. The final part of this paper will examine how hegemonic masculinity was also performed by women, exploring the implications of these blurred performances for understandings of masculinity. Notably, constraints of the paper do not make in depth analysis of the women in Love Island possible, thus, the following interpretation aligns with the methodological perspectives adopted throughout the paper, with alternative analysis possible for future discussion.

Within the 2018 series a contestant called Megan Barton garnered attention for behaving in ways deemed to break the “girl code.” Utilizing a Durkheimian and Goffmanian lens, this refers to deviation from the moral norms and unspoken rules which underpin social order (Goffman, 1963; Durkheim, 2013). Within the example, this refers to when a female begins a relationship or has intimate interactions with a male that her friend is already “coupled up” with. In the latest series, Wes was initially “coupled up” with Laura. This relationship ended abruptly when Megan initiated a relationship with Wes, doing so without consulting Laura about her planned actions. As the premise of the show is to be in a couple, with those not “coupled up” being evicted, arguably Megan was undertaking actions necessary to remain in the villa. However, her behavior was criticized both within the villa by both female and male contestants, as well as by audiences. Her behavior was framed as “unexpected” and unacceptable, as this challenged expectations of female behaviors and femininity. Applying this to sociological thinking, the discomfort and surprise shown toward Megan's behaviors was due to the fact that Megan was blurring the lines of the gender order, performing hegemonic characteristics and “expected” male behaviors (Connell, 2005; Wellard, 2009).

Through her pursuit of Wes, Megan conveyed behaviors which aligned with performances of masculinity associated with the DBS; she was assertive, clinical, and emotionless in her actions, directly contrasting with notions of femininity conveyed in the villa by other women. Through her actions it can be argued that she was performing what Schrock and Schwalbe (2009) refer to as “manhood acts” and, in doing so, her behavior in the villa challenged binaries of masculinity and femininity. Importantly, Megan was displaying what Goffman (1974) refers to as “role distance,” as she was resisting the social structures and expectations via her actions, however, her agency remained curbed by the social structures, evidenced in the judgment from other contestants within the villa (Goffman, 1959, 1974; Trevino, 2003). Budgeon (2013) argues that any shift in norms associated with one side of the binary, affects the social construction of gender beyond the individual. Whilst the example shows that Megan was performing masculinity according to the script of existing gender structures within the villa, the fact that she self identifies as a female challenged the social construction of gender within the space (Yang, 2014). Thus, her performances of “manhood acts” ruptured the gender roles, her agency and actions once again showing the potential for diverse performances of masculinity.

Lorber (1994) suggests that there is a common sense view within society that men and women are naturally different, and that if this binary is to be challenged, then the gender ideologies which structure everyday lives begin to be unraveled. Megan can be seen to be challenging dominant ideologies of gender within the space and this set a precedent in the villa for other deviations from expected gendered behaviors. As the series progressed, viewers saw other women perform behaviors expected of the men (Wellard, 2009). A further example is the contestant Georgia Steel and her interactions with Jack Fowler. Jack and Georgia went on a date, this was despite the fact that Jack was already “coupled up” with Laura, however the premise of the show means that if you are requested to go on a date you must do so, regardless of your relationship status. The two islanders spent time together external to the villa, enabling discrete interactions without the watchful eye of other contestants. The date became controversial and a point of contention for the remainder of the series, as at the end of the date, Georgia attempted to kiss Jack, which she later denied, repeatedly protesting her innocence and declaring her “loyalty” to other women and friendships. Both fellow contestants and the public were outraged by this behavior, branding Georgia a “snake” due to her devious actions. Significantly, Georgia initially denied that this interaction had occurred, with the truth being disclosed by producers later in the series. Similarly to the case of Megan outlined above, Georgia's behavior was seen to be particularly problematic, as it directly challenged notions of gender in the space, rupturing the gender order.

A Butleresque lens on performativity helps to understand examples, such as Megan and Georgia further. Butler (2008) asserts that performances are productive rather than expressive, with a focus on discourse and language, whereby repeated discourses become normalized. This conceptualization of performativity adds to notions of performance outlined so far, in that it is not just the act of performing actions associated with men which meant that Megan and Georgia were viewed as “masculine,” rather, it was the repetition of these which produced the framing of gender and subsequent blurring of the gender order (Salih, 2004; Brickell, 2005; Butler, 2008). The work of Brickell (2005: p. 32) supports this analysis, as he notes that the masculine self is reflexively constructed within performances and that “performances construct masculinity rather than merely reflect its pre-existence.” Applying these ideas together, it is evident that via undertaking actions associated with the DBS and hegemonic masculinity within the villa, these women were not only transcending the expectations of the gender order, rather they were reconfiguring it. Megan and Georgia demonstrated that hegemonic masculinity was evolving and fragmenting. Connell and Messerschmidt (2005) in their reworking of the concept of hegemonic masculinity acknowledge that it has the capacity to adapt over time. Megan and Georgia show that such reformulation of hegemonic masculinity occurs in unexpected ways, with performances of masculinity via females demonstrating that more diverse gender configurations are developing.

## Conclusion

Gender is intertwined in representational, interactional and social process, with television and media culture playing a significant role in the construction of gendered identities (Beynon, 2002; Carter and Steiner, 2004; Brickell, 2005; Gill, 2007; Budgeon, 2013). As reality TV shows, such as Love Island continue to exist and perpetuate heterosexual ideologies, glorifying gendered behaviors and hierarchies, there will be a continuation of a typology of masculinity which aligns with the hegemonic model outlined by Connell (2005). However, analysis of the behaviors and interactions of men within Love Island has shown that diverse masculinities are performed, however, they continue to be softened or minimized within this genre. Significantly, this paper has shown that specific typologies, such as the hegemonic man are problematic, as they do not account for the nuances of men's experiences or divergences from the hegemonic model. Through exploration of choice and resistance, this paper has attempted to capture this nuance, suggesting that men are not struggling to show more diverse facets of their masculine identities; rather such divergences are overlooked. In doing so, this analysis aligns with work on inclusive masculinity theorizing, suggesting that the landscape of masculinity is changing (Anderson, 2012; Magrath, 2017; Anderson and McCormack, 2018).

Via the exploration of masculinity in Love Island the complexity of hegemonic masculinity theorizing, specifically the interplay of hegemonic, subordinated, complicit and marginalized forms of masculinity has been exemplified, with limitations and relevance critically debated (Hearn, 2004; Anderson and McCormack, 2018). Stephen Whitehead (1999:58) asserts that:

“*The concept of hegemonic masculinity goes little way towards revealing the complex patterns of inculcation and resistance which constitute everyday social interaction… it is unable to explain the variant meaning attached to the concept of masculinity at this particular moment in history.”*

The analysis of masculinity in Love Island presented within this paper shows the enduring relevance of this quotation, pointing to the continued empirical limitation of the theory, as well as the necessity of continuing to think critically to encapsulate men's everyday experiences and diverse identities. Following on from Hearn's (2004) discussion of the relevance of hegemonic masculinity theorizing, this paper has shown that men are both a social category formed by the gender system and individuals who have the capacity for agency within social practice. Thus, whilst the concept of hegemonic masculinity should be utilized with caution, it remains a relevant tool in order to understand the lives of men, with further research on the continuity and change in the relationship both amongst men and between men and women in relation to their masculinities needed.

This paper has extended work of thinkers, such as Patulny et al. (2017), through advocating that there is a flow and continuity between different masculine performances (Robinson and Hockey, 2011; Yang, 2014). Whilst a detailed account of the various ways that this unfolds is not possible here, due to the exact focus and constraints of the paper, the analysis presented demonstrates that via the study of transitions between performance there is potential for new understandings of masculinity to be uncovered. Such new directions will enable the complexity and nuance of performances of masculinity, as well as divergence from dominant gender structures to continue to be explored. Furthermore, though this paper discusses local forms of hegemonic masculinity constructed via face-to-face interactions within the villa, the nature of reality TV, such as this, coupled with the myriad ways that audiences engage and interact with this and other forms of media, indicates a continued connection between local and global theorizing on masculinities (Connell and Messerschmidt, 2005; Lusher and Robins, 2009; Messerschmidt, 2012). This highlights the value and relevance of analyzing hegemonic masculinity in various settings and the opportunities for continued reformulation of the concept (Connell and Messerschmidt, 2005).

## Data Availability Statement

The raw data supporting the conclusions of this manuscript will be made available by the authors, without undue reservation, to any qualified researcher.

## Author Contributions

The author confirms being the sole contributor of this work and has approved it for publication.

### Conflict of Interest

The author declares that the research was conducted in the absence of any commercial or financial relationships that could be construed as a potential conflict of interest.

## Footnotes

^1^“Movember” is a UK based charity working to change the face of men's health in the UK.

^2^“CALM” Campaign Against Living Miserably is a movement against suicide amongst men in the UK.

^3^Brah is the informal word used to describe a male friend.

^4^There is not sufficient space to explore Toxic Masculinity within this paper, however it is being utilized here to refer to characteristics of masculinity which are deemed to be harmful or damaging to men.
